# *Lactobacillus plantarum* DK119 as a Probiotic Confers Protection against Influenza Virus by Modulating Innate Immunity

**DOI:** 10.1371/journal.pone.0075368

**Published:** 2013-10-04

**Authors:** Min-Kyung Park, Vu NGO, Young-Man Kwon, Young-Tae Lee, Sieun Yoo, Young-Hee Cho, Sung-Moon Hong, Hye Suk Hwang, Eun-Ju Ko, Yu-Jin Jung, Dae-Won Moon, Eun-Ji Jeong, Min-Chul Kim, Yu-Na Lee, Ji-Hun Jang, Joon-Suk Oh, Cheol-Hyun Kim, Sang-Moo Kang

**Affiliations:** 1 Department of Human Nutrition and Food Science, Chungwoon University, Namjang-Ri, Hongsung-Eup, Hongsung-Kun, Chungnam, Korea; 2 Center for Inflammation, Immunity & Infection, and Department of Biology, Georgia State University, Atlanta, Georgia, United States of America; 3 Department of Animal Resource Science, Dankook University, Dandae-ro, Dongnam-gu, Cheonan-si, Chungnam, Korea; 4 Tobico Inc. Chungnam Techno Park, Jiksan-Eup, Seobuk-Gu, Cheonan-Si, Chungnam, Korea; 5 Department of Microbiology and Immunology, Emory University School of Medicine, Atlanta, Georgia, United States of America; University of Tennessee Health Science Center, United States of America

## Abstract

*Lactobacillus plantarum* DK119 (DK119) isolated from the fermented Korean cabbage food was used as a probiotic to determine its antiviral effects on influenza virus. DK119 intranasal or oral administration conferred 100% protection against subsequent lethal infection with influenza A viruses, prevented significant weight loss, and lowered lung viral loads in a mouse model. The antiviral protective efficacy was observed in a dose and route dependent manner of DK119 administration. Mice that were treated with DK119 showed high levels of cytokines IL-12 and IFN-γ in bronchoalveolar lavage fluids, and a low degree of inflammation upon infection with influenza virus. Depletion of alveolar macrophage cells in lungs and bronchoalveolar lavages completely abrogated the DK119-mediated protection. Modulating host innate immunity of dendritic and macrophage cells, and cytokine production pattern appeared to be possible mechanisms by which DK119 exhibited antiviral effects on influenza virus infection. These results indicate that DK119 can be developed as a beneficial antiviral probiotic microorganism.

## Introduction

Influenza is a serious respiratory disease causing over 220,000 hospitalizations and approximately 36,000 annual deaths in the United States during seasonal epidemics [1]. Vaccination and use of anti-influenza drugs have been undertaken worldwide. However, current vaccines are only effective if they are well matched with the circulating influenza viruses. A recent pandemic outbreak of the new 2009 H1N1 virus provides a good example of a limited efficacy of current vaccination [2]. Antiviral medications also have limitations such as immediate administration after infection, adverse reactions, and emergence of drug resistant strains [3]–[7]. Thus, it is desirable to find a more general measure that would have protective effects on influenza virus.

Probiotics are micro-organisms which bestow health benefits on the host when administered in adequate amounts. The most common probiotics include lactic acid bacteria. Various fermented vegetables and dairy products contain a variety of lactic acid bacteria that were shown to provide health benefits and improvements to a certain level [8]–[11]. The capability of lactic acid bacterial species to grow in low pH and/or high salt conditions in fermented foods enables lactic acid bacteria to survive gastric acid in the stomach and bile acid in the intestines [12], [13]. Fermented foods are a good source for isolating various beneficial lactic acid bacterial strains. It was demonstrated that probiotics of some lactic acid bacterial strains were able to protect against infectious diseases [14], [15], and have the anti-allergic effects on immune diseases in mice [16], [17] and humans [18], [19]. Yogurt fermented with *Lactobacillus* was shown to reduce the cases of catching cold in the healthy elderly [20] and to prolong the survival periods of mice with influenza virus infection [21]. In particular, previous studies demonstrated the protective effects on subsequent influenza virus infection by administration of various lactic acid bacterial strains via the oral route [22]–[25] or the intranasal route [15], [26]–[29]. In previous studies, the effects of lactic acid bacteria on influenza include partial survival protection or prolonged survival periods. It remains unknown whether treatments with lactic acid bacteria can confer improved protection by reducing weight loss of infected animals and thus ameliorating morbidity.

In the present study, we determined whether the *Lactobacillus plantarum* DK119 (DK119) newly isolated from the fermented vegetable food “Kimchi” would confer enhanced protection against the subsequent influenza virus infection via the intranasal or oral administration. The antiviral efficacy of DK119 was evaluated by monitoring weight changes, and examining lung viral loads and histology. We further tested the hypothesis that modulating cytokine responses and innate immune cells would play an important role in providing protection against influenza virus by DK119 as a beneficial probiotic. Here, we also found that alveolar macrophage cells were critically important in mediating protection conferred by DK119.

## Results

### Intranasal Administration of Mice with DK119 Confers Protection Against Lethal Infection

**Figure 1:**
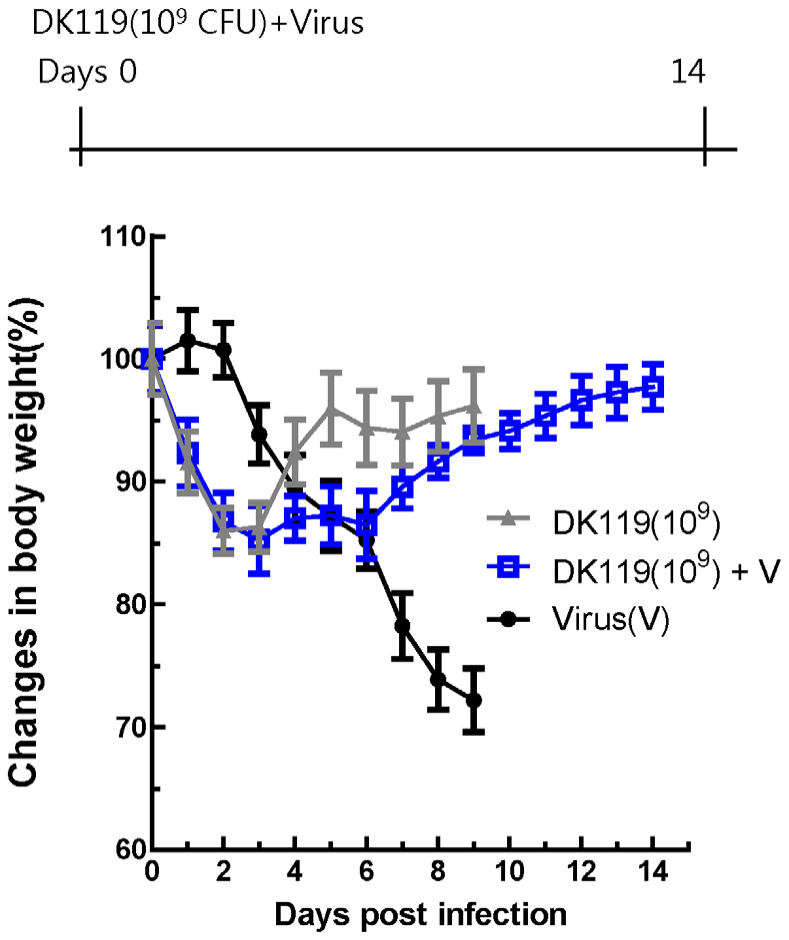
Intranasal co-administration of DK119 (10^9 ^CFU) and A/PR8 H1N1 virus confers protection. BALB/c mice (n = 5 per group) were infected with A/PR8 virus (2.5 LD_50_) alone or co-administered with DK119 (10^9^ CFU per mouse) and A/PR8 H1N1 virus, and body weight changes were monitored daily for 14 days. Virus (V): mice infected with A/PR8 virus, DK119(10^9^)+V: mice infected with DK119 and A/PR8 H1N1 virus.DK119 (10^9^): mice treated with DK119 (10^9^ CFU per mouse) alone. Bars indicate standard errors.

**Figure 2:**
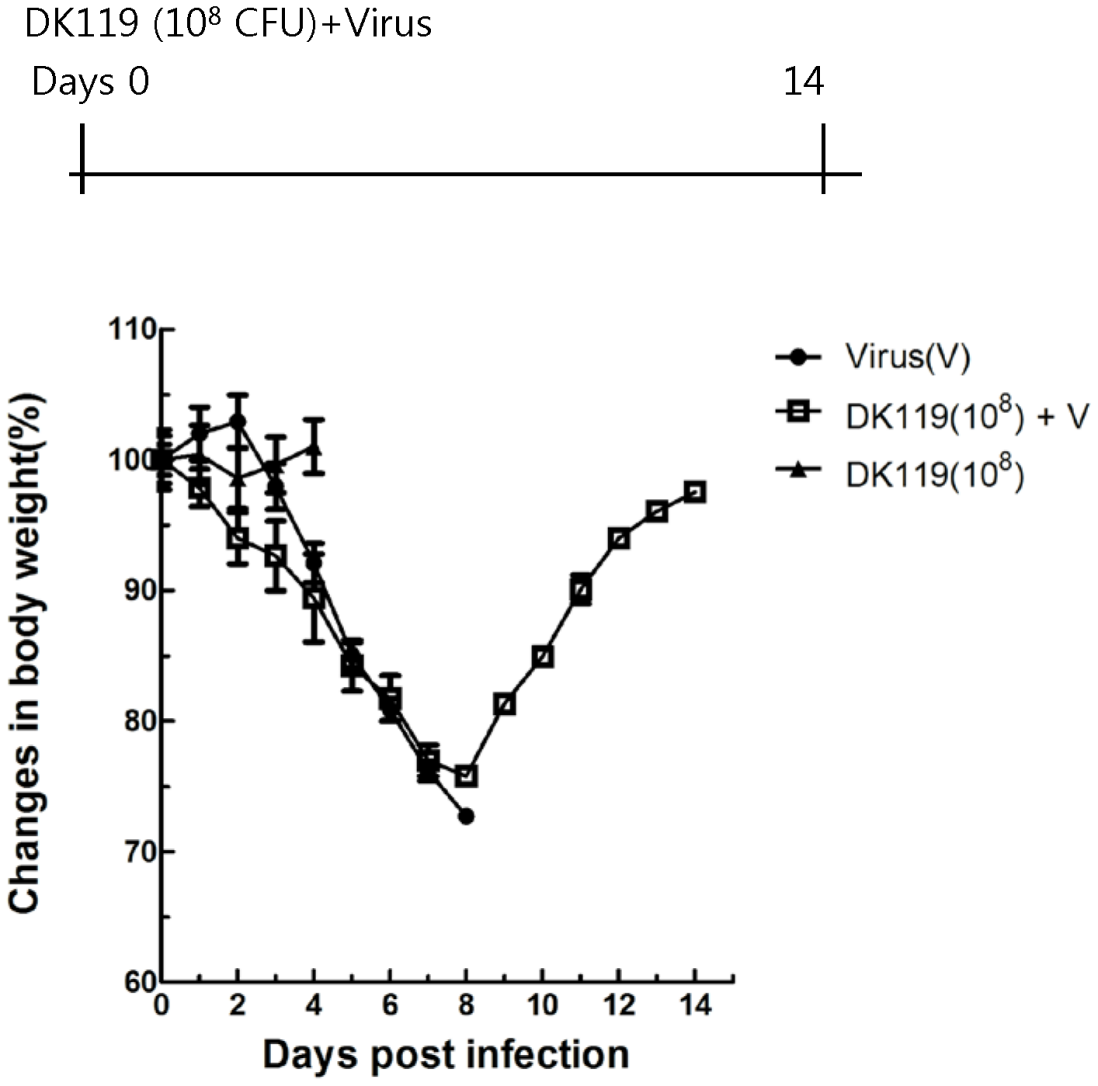
Intranasal co-administration of a low dose of DK119 (10^8 ^CFU) and A/PR8 virus (H1N1) provides survival protection. BALB/c mice (n = 6 per group) were infected once with A/PR8 virus (2.5 LD_50_) alone or co-administered with DK119 (10^8^ CFU per mouse) and A/PR8 virus, and monitored daily for 14 days. Virus(V): mice infected with A/PR8 virus, DK119 (10^8^)+V: mice infected with DK119 and A/PR8 virus.DK119 (10^8^): mice treated with DK119 (10^8^ CFU per mouse) alone did not show weight losses.

Respiratory tracts and lung are major tissues for influenza viral replication. To determine the local protective effects of DK119 after local treatment, we carried out intranasal administration of DK119 and influenza virus together. A group of mice was intranasally infected with influenza virus only or influenza virus mixed with DK119. Mice that were infected with the influenza virus (2.5 LD_50_) and DK119 (10^9^ CFU per mouse) showed initial weight loss of approximately 10 to 15% but all recovered their weights to the normal level (Fig. 1A, B). However, all mice in DK119 untreated group were very sick as determined by their activity and weight loss, and died or had to be euthanized after infection with the same dose of influenza virus. The weight loss at early time points (Fig.1A) was probably because of a high dose of DK119 administered intranasally (10^9^ CFU/mouse) since the DK119 only treated control group showed a transient loss in body weight of mice (Fig. 1). To further determine the dosage effects of DK119 on providing protection against influenza virus, a low dose of DK119 (10^8^ CFU per mouse) was tested similarly (Fig. 2). The body weight loss was found to be less at the time of day 2 post infection compared to the high dose treatment although the overall weigh loss was severe, resulting in over 20% loss at day 8 post infection. Also, the survival protection was observed to be 50% in the group treated with a low dose of DK119 (10^8^ CFU/mouse). The untreated control group showed no protection after influenza virus infection. These results suggest that DK119 treatment has an anti-viral effect on influenza virus infection in a dose-dependent manner.

### Effects of Pretreatment of Mice with DK119 on Influenza Virus Infection

**Figure 3:**
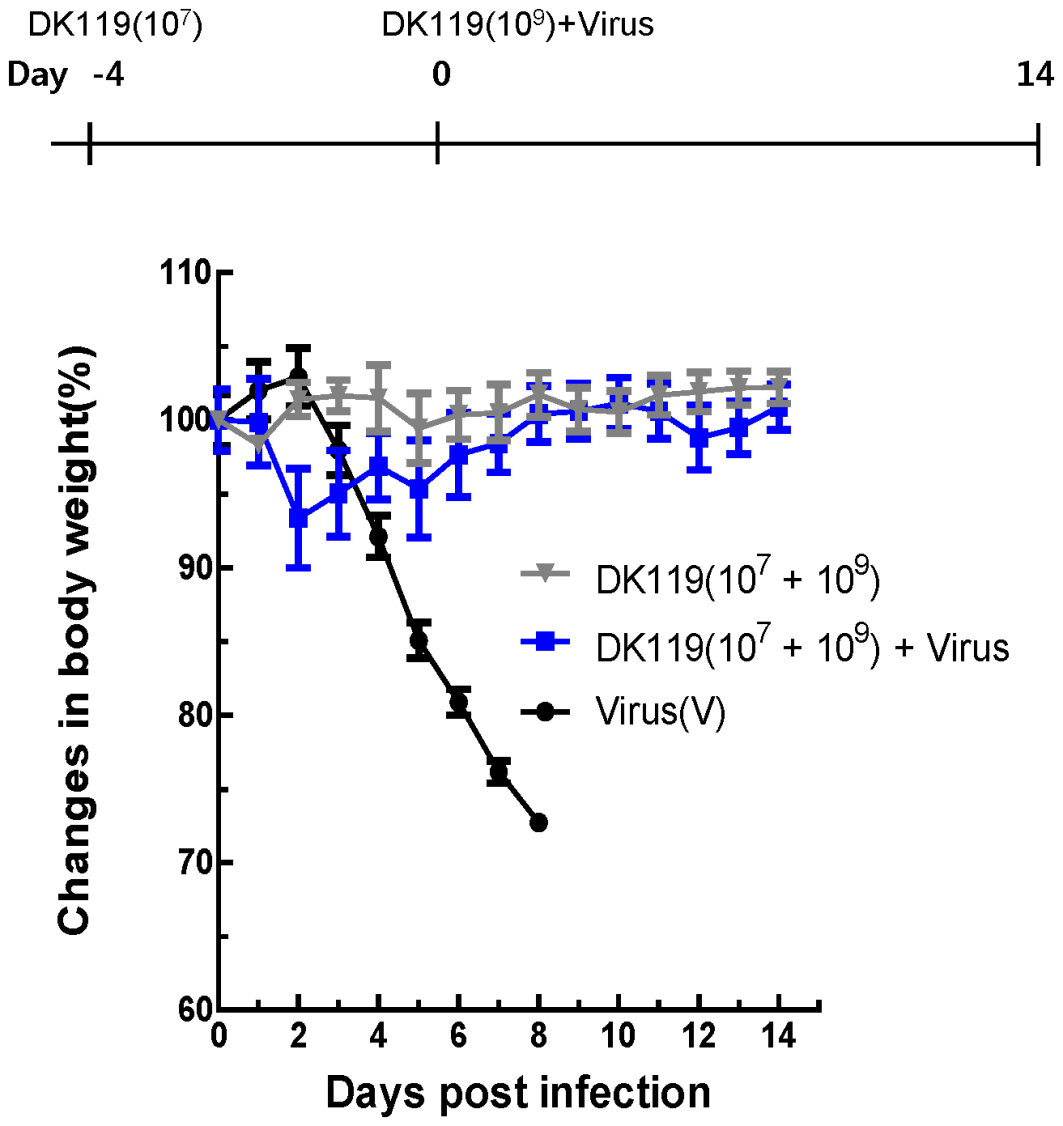
Pretreatment of a low dose of DK119(10^7^ CFU) confers improved protection by preventing severe weight loss. A group of mice (n = 6) was once treated intranasally with a low dose of DK119 (10^7^ CFU per mouse). After 4 days of DK119 treatment, mice were infected with A/PR8 H1N1 virus (2.5 LD_50_) in the presence of DK119 (10^9^ CFU per mouse). The control group was infected with A/PR8 virus without DK119 and no pretreatment. Virus(V): mice infected with A/PR8 H1N1 virus, DK119(10^7^+10^9^)+V: mice pretreated with DK119 and infected with DK119 and A/PR8 H1N1 virus.DK119(10^7^+10^9^): mice treated with DK119 (10^7^ CFU and 10^9^ CFU per mouse). Bars indicate standard errors.

Pretreatment of mice with other lactic acid bacteria was shown to provide survival benefits after influenza virus infection [15], [29]. Therefore, we hypothesized that pretreatment with DK119 would improve the protective efficacy. Mice were once treated with a low dose of DK119 (10^7^ CFU/mouse) 4 days earlier prior to infection. Then, mice were infected with a lethal dose of influenza virus in the presence of DK119 (10^9^ CFU/mouse) (Fig. 3). All mice with intranasal pre-treatment of DK119 showed only minimum (∼5–7%) loss of weight and survived the lethal infection, indicating that protection by DK119 treatment was significantly improved. It is also important to note that pretreatment with a low dose of DK119 (10^7^ CFU/mouse, Fig. 3) prevented even the transient loss of body weight that was observed after high dose treatment with DK119 alone (10^9^ CFU/mouse, Fig. 1). Therefore, pretreatment with DK119 prior to infection significantly improved protection against influenza virus (Fig. 3).

**Figure 4:**
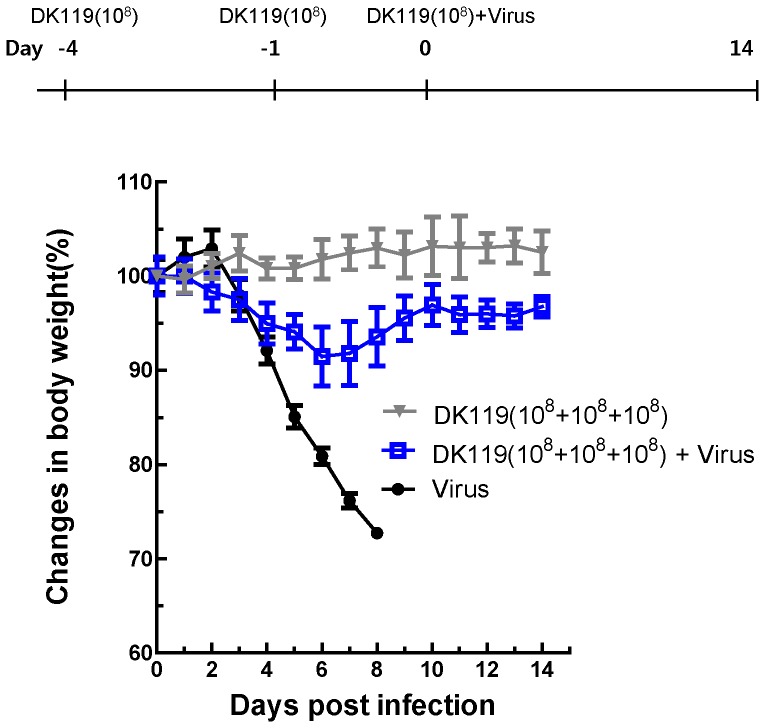
Effects of DK119 (10^8^ CFU) treatment on preventing weight loss against influenza H1N1 virus infection. A group of mice (n = 6) was treated with DK119 (10^8^ CFU/mouse) at 4 days and 1 day prior to infection with A/PR8 virus (2.5 LD_50_) in the presence of a low dose of DK119 (10^8^ CFU per mouse). Virus(V): mice infected with A/PR8 virus, DK119 (10^8^+10^8^+10^8^)+V: mice pretreated twice with DK119 and infected A/PR8 H1N1 virus in the presence of with DK119 (10^8^ CFU per mouse). Bars indicate standard errors.DK119 (10^8^+10^8^+10^8^): mice treated with DK119 alone (10^8^ CFU/mouse at 3 different time points without virus infection).

The dose of DK119 (10^9^ CFU/mouse) tested above appeared to be high and use of a low dose would be desirable. To determine whether a low dose of DK119 (10^8^ CFU/mouse) would be protective, mice were treated with DK119 (10^8^ CFU/mouse) two times at days 4 and 1 prior to lethal infection with influenza virus (A/PR8 virus) in the presence of a10 fold low dose of DK119 (10^8^ CFU/mouse) (Fig. 4). DK119 alone treatment did not affect mouse body weight changes (Fig. 4). All mice with intranasal DK119 pretreatment were 100% protected against lethal infection of influenza virus only a moderate level of body weight loss (6–9% loss) at day 6–7 post infection (Fig. 4). In support of our hypothesis, the pretreatment with DK119 prior to infection showed improved protection by preventing substantial weight loss even at the low dose of DK119 (10^8^ CFU/mouse) compared to the severe weight loss observed in mice infected with influenza virus and the same dose of DK119 (Fig. 2).

### Effects of Oral Administration with DK119 on Influenza Virus Infection

**Figure 5:**
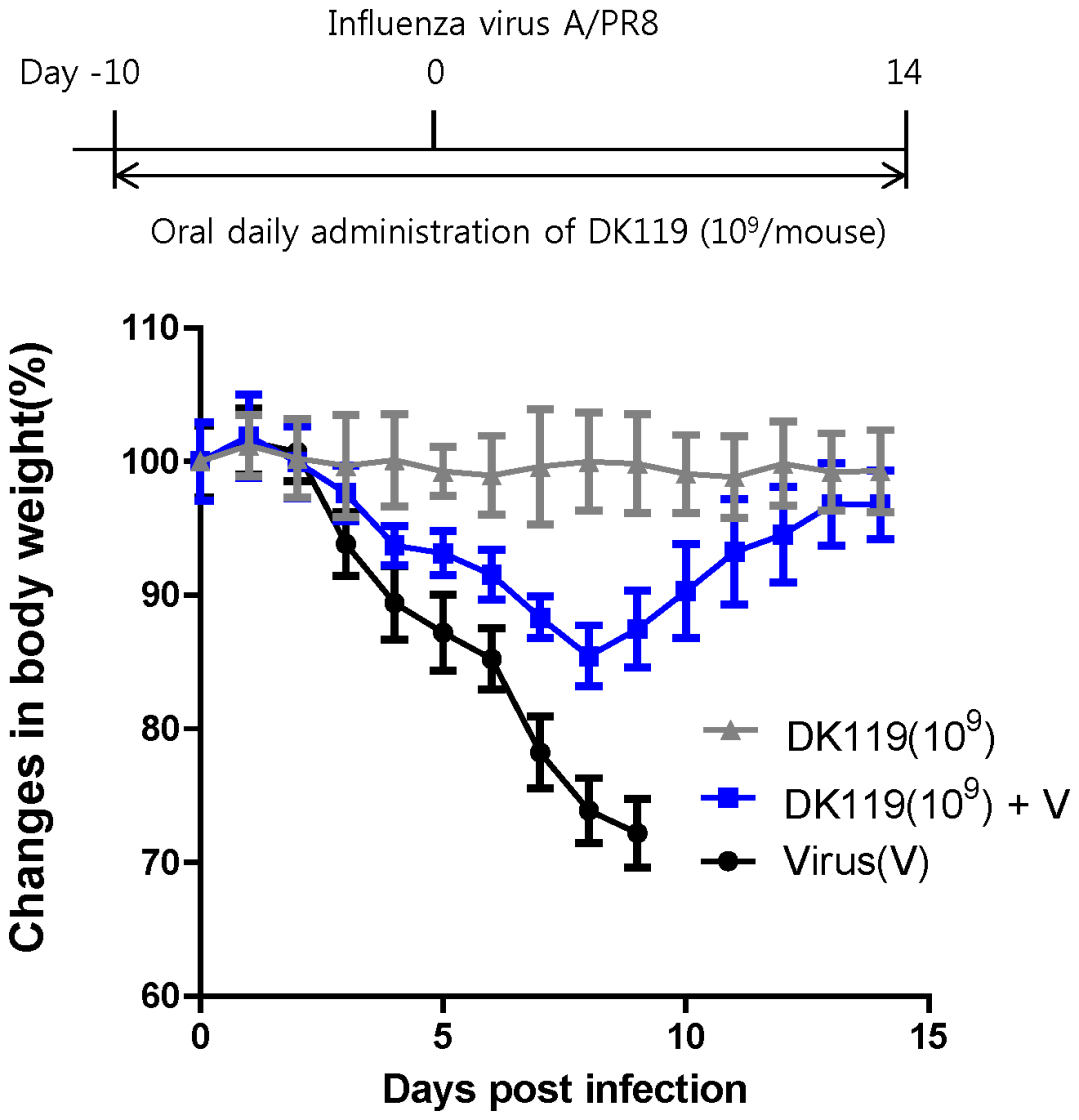
Oral administration of DK119 (10^9^ CFU) has protective effects against influenza virus infection. Mice (n = 5) were administered DK119 (10^9 ^CFU/mouse daily) orally for 10 days before infection and for 14 days after infection with A/PR8 H1N1 virus (2.5 LD_50_), and their body weight changes were recorded. Virus(V): A/PR8 virus infection, DK119(10^9^)+V: Oral administration with DK119 and then virus infection. Bars indicate standard errors. DK119(10^9^): mice treated orally with DK119 alone (10^9^ CFU/mouse daily for 14 days).

**Figure 6:**
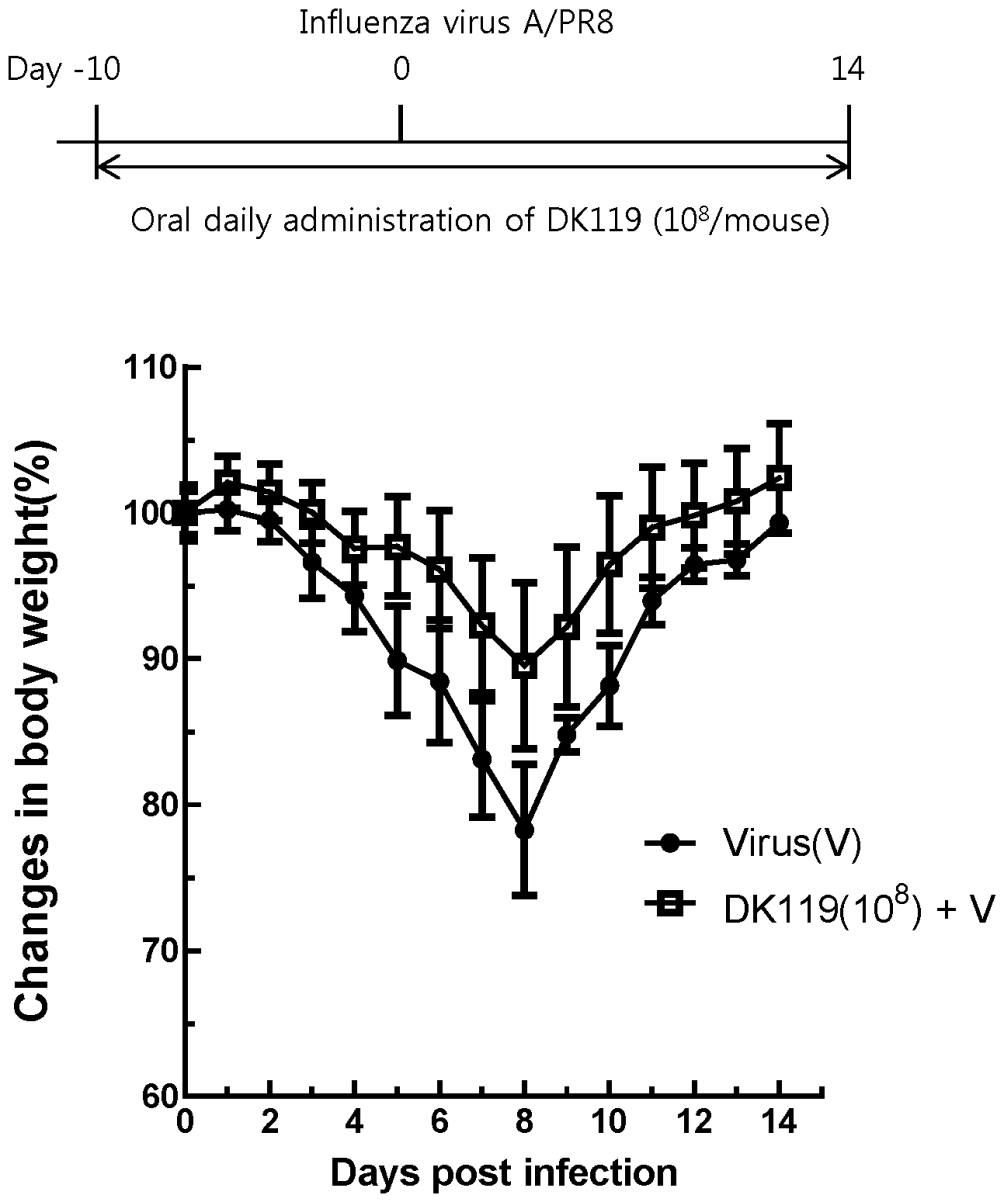
Oral administration of mice with a low dose of DK119 has protective effects against influenza virus (H1N1) infection. Mice (n = 6) were orally administered DK119 (10^8 ^CFU/mouse daily) for 10 days before infection and for 14 days after infection with A/PR8 virus (1 LD_50_). Body weight changes were recorded. Virus(V): A/PR8 virus infection, DK119 (10^8^)+V: Oral administration with DK119 and virus infection.

Fermented foods that contain DK119 are orally consumed in humans, and thus the effects of oral administration with DK119 would be a more appropriate route to be tested. Mice (n = 6 per group) were orally administered DK119 (10^9^ CFU/mouse) daily for 10 days prior to infection with influenza virus. After infection with influenza H1N1 virus (A/PR8, 2.5 LD_50_), DK119 oral administration daily was continued for 14 days. Untreated mice showed over 25% weight loss and had to be euthanized (Fig. 5). The DK119 treated group showed a moderate weight loss approximately 14% at peak and then 100% mice survived. A lower dose of DK119 (10^8^ CFU/mouse) via oral administration also conferred 100% survival protection against a low dose (1 LD_50_) of influenza A/PR8 virus infection (Fig. 6). These results indicate that DK119 oral administration provides beneficial effects on protecting influenza virus infection in spite of a certain degree of morbidity.

### DK119 Treatment Lowers Lung Viral Loads after Influenza Infection

**Figure 7:**
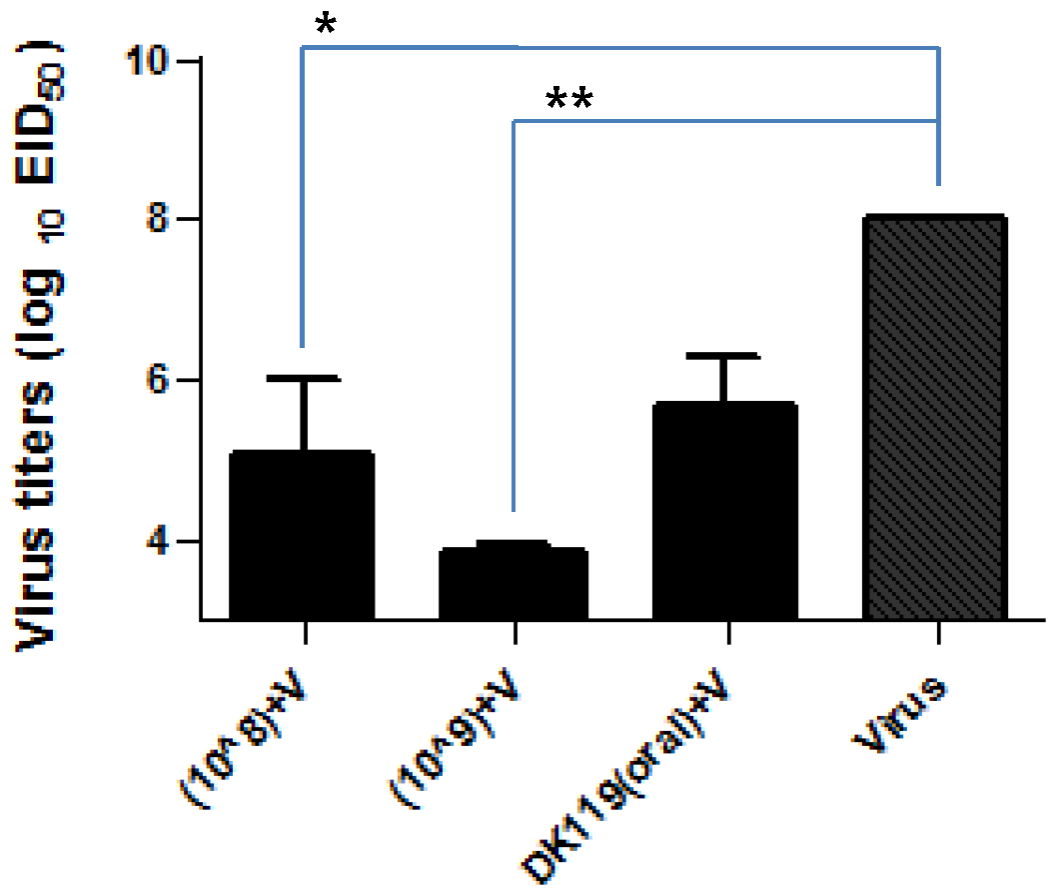
DK119 pretreatment promotes lung viral clearance after influenza virus infection. Lung viral titers at day 4 post infection with H1N1 influenza virus A/PR8 are determined and presented in log10 units of limiting dilutions showing viral replication in chicken eggs. (10?8)+V: mice intranasally pretreated with DK119 (10^8^ CFU) and infected with A/PR8 virus in the presence of DK119 (10^8^). (10?9)+V: mice intranasally pretreated with DK119 (10^7^ CFU) and infected with A/PR8 virus in the presence of DK119 (10^9^). DK119 (oral)+V: mice orally pretreated with DK119 (10^9^ CFU) for 10 days and then infected with A/PR8 virus. Virus: Naïve mice infected with A/PR8 virus (2.5 LD_50_). * (p<0.05), ** (p<0.001) statistical significances are indicated. Bars indicate standard errors.

Viral loads in the lungs provide important information for assessing the efficacy of protection against influenza virus infection. At day 4 post infection with H1N1 influenza virus, lung samples were collected and viral titers were determined as an egg infectious dose (Fig. 7). Lung viral load was detected at a high level in mice with influenza virus infection in the untreated control (Fig. 7). DK119 oral administration (10^9^ CFU/mouse) or intranasal treatment (10^8^ CFU/mouse) resulted in a reduction of lung viral loads by approximately 200 to 800 fold (Fig. 7). Interestingly, lung viral load was significantly reduced to more than a 4 log lower level in the group of mice that were intranasally treated with a high dose DK119 (10^9^ CFU/mouse) during infection compared to that in the untreated control group (Fig. 7). Therefore, DK119 oral or intranasal administration can reduce lung viral loads in mice after influenza virus infection.

### DK119 Treatment Increases the Production of Cytokines IFN-γ and IL-12

**Figure 8:**
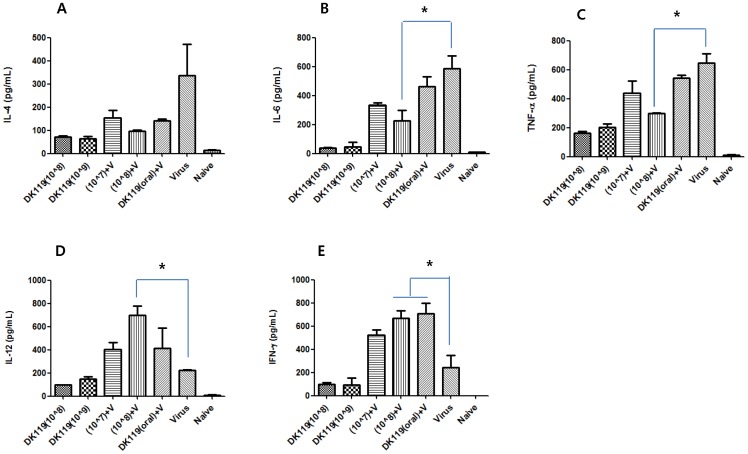
Effects of DK119 pretreatment on cytokine production upon influenza virus infection. Levels of cytokines in bronchoalveolar lavage fluids (BALF) collected from mice (n = 3) at day 4 post infection with A/PR8 virus are presented in pg/ml as determined using cytokine ELISA. (A) IL-4, (B) IL-6, (C) TNF-α, (D) IL-12, (E) IFN-γ. DK119 (10?8): Intranasal DK119 10?8 control in the absence of A/PR8 virus infection. DK119 (10?9): Intranasal DK119 10?9 control in the absence of A/PR8 virus infection. (10?7)+V: DK119 (10?7) intranasal pretreatment and then A/PR8 virus infection in the presence of DK119 (10?9). (10?8)+V: DK119 (10?8) intranasal pretreatment and then A/PR8 virus infection in the presence of DK119 (10?9). Virus: Naïve mice infected with A/PR8 virus (2.5 LD_50_). * indicates statistical significance compared to the control virus group (p<0.05). Bars indicate standard errors.

**Figure 9:**
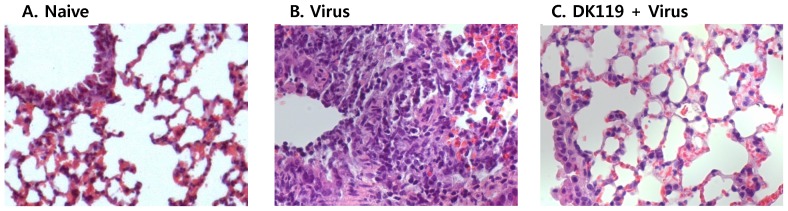
DK119 pretreatment reduces influenza virus-induced lung histopathology. Inflammatory histopathology after H&E staining of lung sections was compared from mice that were infected with influenza A/PR8 virus (H1N1) with or without DK119 treatment. (A) Naïve: Naïve mice without A/PR8 virus and DK119, (B) Virus: mice that were infected with A/PR8 virus without DK119 treatment, (C) DK119+Virus: DK119 (10^7^) intranasal pretreatment and then A/PR8 virus infection in the presence of DK119 (10^9^). All images were obtained using the same camera setting and shown at a magnification of X400.

Inflammatory cytokines are known to be associated with severe lung disease and high mortality. To better understand beneficial effects by DK119 treatment, cytokine levels were determined in bronchoalveolar lavage fluids (BALF) at day 4 post infection. DK119 treated mice (10^8^, 10^9^, Fig. 8) showed low levels of cytokines compared to the virus infected mice without DK119 pretreatment. Levels of cytokines IFN-γ and IL-12 were found to be elevated in BALF from mice orally or intranasally treated with DK119 compared to those from mice that were infected with influenza virus without DK119 (Fig 8D, E). The DK119 intranasally administered group (10^8^+V, Fig. 8) showed the highest level of IL-12 and lower levels of IL-4 and IL-6 cytokines. In particular, IFN-γ was also found to be high in sera and lung extracts from DK119 treated mice upon influenza virus infection (data not shown). In contrast, cytokines IL-4, IL-6, TNF-α were detected at lower levels in BALF from mice with DK119 treatment compared to those from mice that were infected with influenza virus without DK119 (Fig. 8). Consistent with the pattern of inflammatory cytokines, a high degree of inflammation was observed in the peribronchial and perivascular alveolar lung tissues from influenza virus infected mice but not from mice with intranasal DK119 pretreatment (Fig. 9). Therefore, it is speculated that DK119 treatment enhances innate immune responses stimulating the production of T helper 1 type (Th1) anti-viral cytokines (IFN-γ, IL-12) and suppressing Th2 like or inflammatory cytokines (IL-4, IL-6, TNF-α) responses.

### Depletion of Dendritic and Macrophage Cells Abolishes Protection Mediated by DK119 Treatment

**Figure 10:**
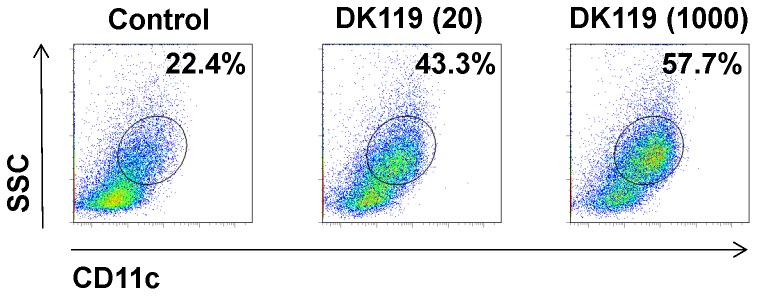
DK119 increases CD11c^+^ cell populations from BMDCs. Bone marrow derived dendritic cells (BMDCs) were seeded in concentrations of 2.5×10^5^ cells/ml and treated with 5×10^6^ CFU/ml of DK119 or 2.5×10^8^ CFU/ml for 2 days. The cells within a circle are CD11c positive cells and their percentages indicated. A representative figure in each is shown from three independent experiments. Control: No DK119, DK119 (20): 5×10^6^ CFU/ml of DK119, DK119 (1000): 2.5×10^8^ CFU/ml of DK119.

For better understanding the effects of DK119 on improving protection, bone marrow derived dendritic cells (BMDCs) were treated with DK119 and analyzed by flow cytometry (Fig. 10). DK119 treatment on BMDCs resulted in increases of CD11c^+^ populations by 2 to 2.5 fold (Fig. 10). Therefore, increases in dendritic cell populations by DK119 might be contributing to improving the protection against influenza virus infection.

**Figure 11:**
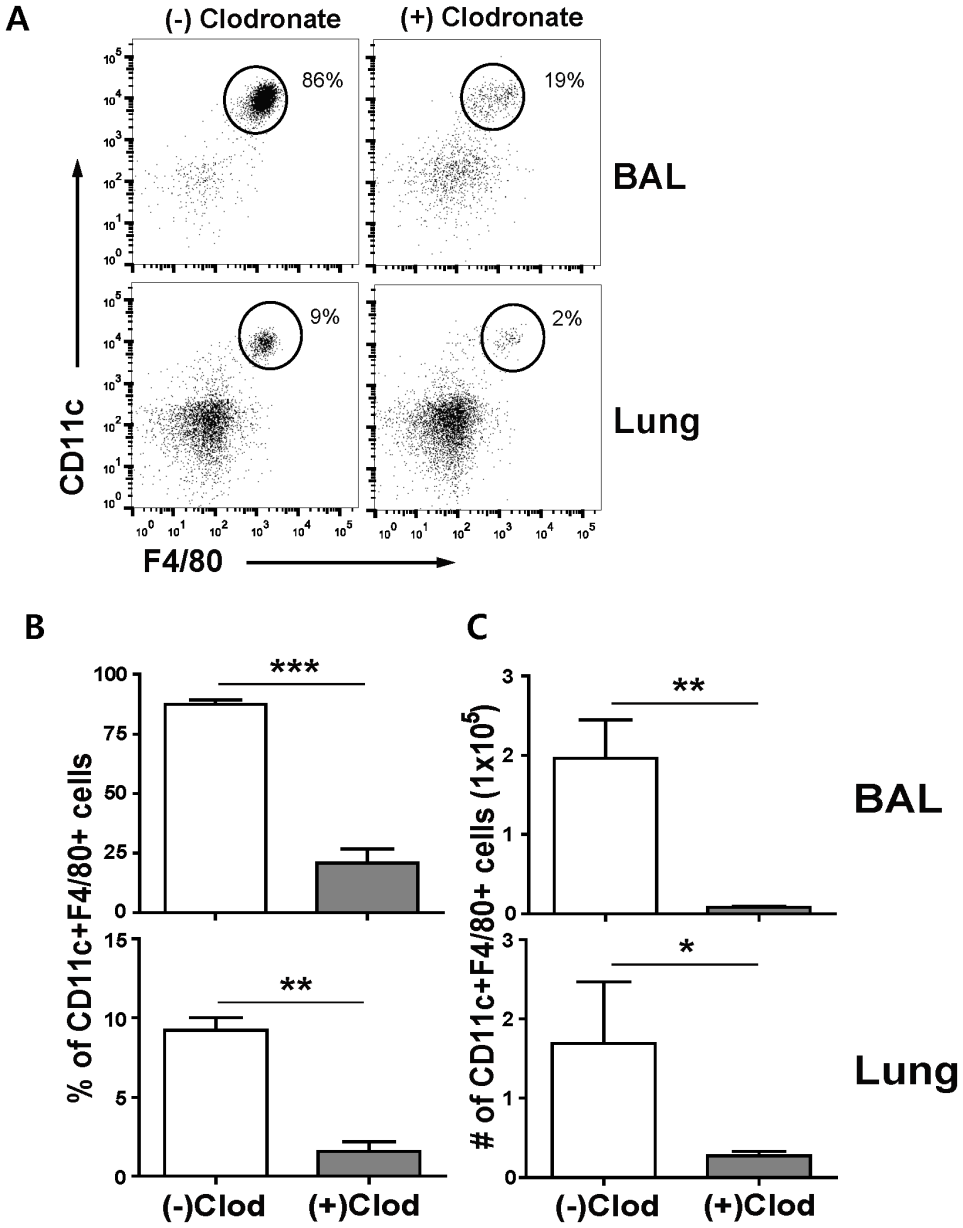
Intranasal administration of clodronate-liposomes selectively depletes CD11c^+^F4/80^+^alveolar macrophages in lungs and bronchoalveolar lavages. The cells from the bronchoalveolar lavage (BAL) and lungs from mice (n = 3) were harvested4 days after clodronate injection. CD3^+^ and CD11b^+^ cells were excluded to determine the effect of clodronate on depleting alveolar macrophages with CD3^−^CD11b^−^CD11c^+^F4/80^+^markers. (A) Representative profiles of flow cytometry gating of CD3^−^CD11b^−^CD11c^+^F4/80^+^phenotypic cells. (B) Percentages of CD3^−^CD11b^−^CD11c^+^F4/80^+^cells. (C) Cellularity of CD3^−^CD11b^−^CD11c^+^F4/80^+^cells. (−) Clodronate or (−)Clod: No clodronate control mice, (+) Clodronate or (+)Clod: Clodronate treated mice. BAL: Bronchoalveolar cells, Lung: Cells collected from lung tissues. *P<0.05, **P<0.001, ***P<0.0001 (Student T-test). Bars indicate standard errors.

Since alveolar macrophages were reported to contribute to controlling lung viral loads and reducing mortality [30], we further hypothesized that lung airway dendritic and macrophage cells would play a vital role in conferring DK119-mediated protection against influenza virus. It was previously reported that dendritic and macrophage cells were selectively depleted by intranasal or intratracheal administration with clodronate-liposomes [31], [32]. F4/80^+^CD11c^+^CD11b^−^ phenotypic cells were classified as alveolar macrophages in a study of infected lungs [33]. We found that intranasal administration of clodronate-liposomes resulted in significant depletion of CD3^−^CD11b^−^CD11c^+^F4/80^+^ phenotypic alveolar macrophage cells in the lungs (Fig. 11). The percentages (Fig. 11B) and cellularity (Fig. 11C) of CD11c^+^F4/80^+^ phenotypic alveolar macrophage cells were found to be depleted by approximately 80% in bronchoalveolar lavages and lungs.

**Figure 12:**
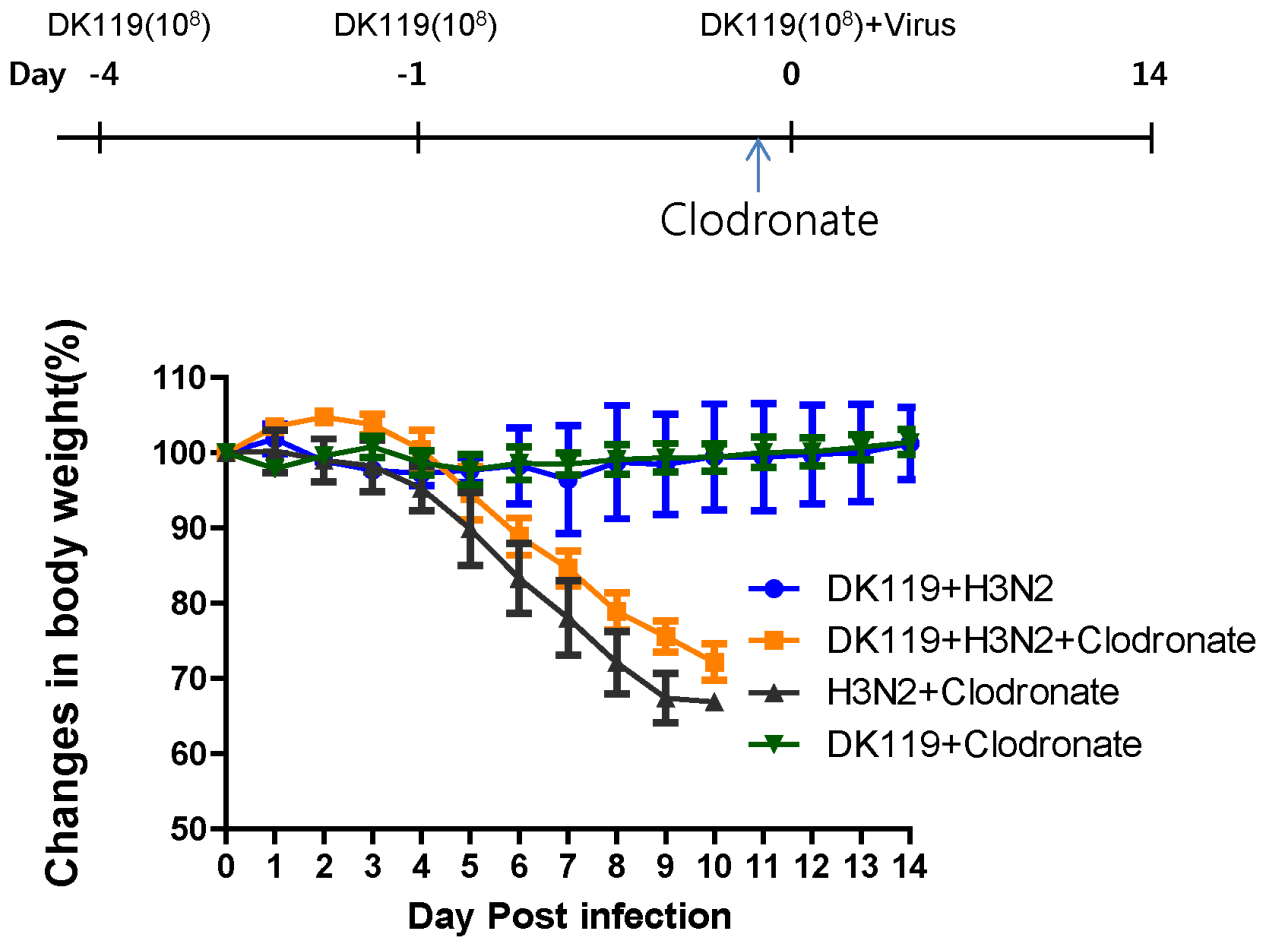
Effects of clodronate treatment on DK119-mediated protection against H3N2 influenza virus infection. Groups of mice (n = 6) were two times treated with DK119 (10^8^ CFU/mouse) at 4 and 1 day earlier, then either received clodronate or PBS 4 hours prior to infection with A/Philippines/82H3N2 virus (2.5 LD_50_) in the presence of a low dose of DK119 (10^8^ CFU per mouse). DK119+H3N2: mice pretreated with DK119 were infected with A/Philippines/82 (H3N2) virus. DK119+clodronate+H3N2: mice pretreated with DK119 were intranasally administered clodronate prior to infection with A/Philippines/82 (H3N2) virus. H3N2+Clodronate: mice treated with clodronate 4 hours prior to infection.DK119+Clodronate: mice treated with DK119 at the same doses and then clodronate administration. Bars indicate standard errors.

A group of mice was treated with DK119 probiotic at a low dose (10^8^ CFU/mouse). To determine the effects of the selective depletion of dendritic and macrophage cells on DK119-mediated protection, DK119 treated mice with or without clodronate-liposome administration were infected with a lethal dose of mouse-adapted influenza A/Philippines/82 H3N2 virus (Fig. 12).DK119 alone treatment even in the administration of clodronate did not affect mouse body weight changes. More importantly, all mice in DK119 treated group without clodronate survived lethal infection and did not exhibit weight loss. It is worth to note that DK119 pretreatment could provide good protection against H3N2 influenza virus. The group of DK119 treated mice that received intranasal administration of clodronate showed severe weight loss and did not show any protection (Fig. 12). These results indicate that alveolar macrophage cells play a critically important role in DK119-mediated protection. In addition, DK119 pretreatment can provide protection against A/Philippines/82 (H3N2) virus, which is similar to the protection against A/PR8 (H1N1) virus.

## Discussion

This study reports that *Lactobacillus plantarum* DK119 has significant antiviral effects on influenza virus infection via intranasal or oral administration, conferring survival protection as well as preventing substantial weight loss. In previous studies reporting antiviral protection, mice treated with different strains of *Lactobacillus* exhibited prolonged survival periods without protection [22], [23] or were only partially protected after lethal infection [25]–[27], but illness has not been reported. It was recently demonstrated that various *Lactobacillus* species showed differential protective efficacies with a wide range of 0–100% survival rates against influenza virus infection depending on administration routes and doses [15]. Thus, in this study, we report substantial antiviral activity of DK119, preventing illness as indicated by diminishing weight losses in mice after lethal infection with influenza viruses as well as conferring 100% survival protection against H1N1 and H3N2 influenza A viruses.

In contrast to most previous studies reporting only survival rates [22], [23], [25]–[27], we determined body weight changes as an important measure of illness, which should be considered for evaluating the protective efficacy. Results in this study suggest that intranasal treatment with low doses of DK119 prior to influenza virus infection significantly improves protection by preventing severe weight losses. Also, the protective efficacy against influenza virus infection was observed in a DK119 dose-dependent manner. Another important parameter in evaluating protective efficacy is a magnitude of controlling lung viral loads. Intranasal or oral treatment of mice with DK119 lowered lung viral titers by 200 to 10000 fold. The efficacy of lowering lung viral titers was dependent on doses and routes of DK119 treatments. Although direct comparison is not possible, previous studies reported approximately 10 to 20 fold decreases in lung viral titers at the day 2–3 post infection [15], [22], [23], [28]. Therefore, it is assumed that DK119 strain as a novel strain of *Lactobacillus* tested in this study might have potent antiviral activity against influenza virus.

Pathogenesis of influenza virus infection is known to be involved with hyper-inflammatory responses [30], [34]–[36]. In particular, levels of cytokines IL-6 and TNF-α were reported to be positively correlated with influenza associated lethality [35], [37]. In this study, mice that were infected with influenza A/PR8 virus showed high levels of inflammatory cytokines, IL-4, IL-6, TNF-α, but low levels of IFN-γ and IL-12. In contrast, DK119 treatment of mice resulted in a reverse pattern of cytokines, increasing the levels of IFN-γ, IL-12,and decreasing inflammatory cytokines (IL-4, IL-6, TNF-α). In particular, IFN-γ was prominently detected in BALF, lung extracts, and sera from DK119-treated mice. Consistent with results in this study, oral administration of *Lactobacillus plantarum* strain was shown to enhance the production of Th1 type cytokine IFN-γ in response to ovalbumin immunization [38]. Similarly, intranasal administration of *Lactobacillus pentosus* strain was previously reported to induce Th1 like cytokines IL-12 and IFN-γ possibly contributing to host resistance to influenza infection [28]. Although the source of cytokine producers is not clear in this study, innate immune cells such as macrophages, dendritic cells, neutrophils, and natural killer cells would be the candidate cells [30]. Innate immunity is the first defense host mechanism in responding to pathogens. Thus, modulating innate immunity would be a mechanism for DK119-mediated protection against influenza. However, detailed mechanisms remain to be determined.

Macrophage and dendritic cells (DCs) play important roles in activating innate and adaptive immune systems by their initial responses to environmental microorganisms. We found that protection conferred by DK119 treatment was completely abolished when alveolar macrophage cells (CD11c^+^F4/80^+^) were depleted in these mice prior to infection with influenza A/Philippines/82 H3N2 subtype virus (Fig. 12). Naïve mice that were infected with the same dose and virus were not protected in the absence of DK119 treatment regardless of clodronate-mediated depletion of alveolar macrophages (Fig. 12). Therefore, it is speculated that DK119 treatment modulates alveolar macrophages and probably dendritic cells in a way for promoting protective host innate immunity to influenza infection. Also, we found thatDK119 induced the substantial expansion of CD11c^+^cells of BMDCs *in vitro*. In support of this hypothesis, neutrophils and alveolar macrophage cells were reported to play an important role in controlling influenza virus replication and enhancing survival of infected mice [30]. It was also reported that *Lactobacillus* stimulated human dendritic cells to secrete Th1/Th17 cytokines including IL-12 [39]. *Lactobacillus* was also shown to induce the production of Th1 polarizing cytokine IL-12 by stimulating murine dendritic cells via Toll-like receptor-2-dependent mechanism [40]. There is a possibility that the capability of DK119 to induce IL-12 and IFN-γ cytokines in innate immune cells such as dendritic, macrophage cells, and neutrophils subsequently contributes to improving an anti-viral immunity of the host. Further studies remain to be performed as a future direction to better understand the DK119-mediated protection against influenza.

In conclusion, administration of DK119 provided protection against infection with H1N1 and H3N2 influenza viruses, probably by enhancing the innate immunity of CD11c^+^ dendritic and macrophage cells and anti-viral cytokines, and thus contributing to better controlling of lung viral loads. DK119 is orally consumed in humans through various fermented cabbage foods and various forms of commercial food products. There might be some concerns about the safety of using DK119 via the intranasal route although apparent adverse effects were not observed in mice with low doses of DK119 (10^7^–10^8^ CFU) pretreatment intranasally. Histology data in this study indicated that intranasal pretreatment with DK119 almost prevented virus-induced lung inflammation (Fig. 9). These results in this study support evidence that further studies on the use of DK119 as a beneficial probiotic will be important.

## Materials and Methods

### Preparation of *Lactobacillus plantarum* DK119 and Virus

*Lactobacillus plantarum* strain DK119 (DK119) isolated from Korean fermented vegetable food “*Kimchi”* was previously described [41]. *Lactobacillus plantarum* DK119 was suggested to be a promising strain as probiotics because it showed tolerance to acid and bile salt [41]. DK119 was cultured in MRS broth (Difco, Detroit, MI) at 37°C for 24 h. Cultured DK119 were harvested by centrifugation at 10,000 rpm at 4°C for 10 minutes, washed twice with phosphate-buffered saline (PBS), and suspended in saline for the following experiments. DK119 colony forming units (CFU) were determined by counting colonies on the MRS agar plates. Mouse adapted influenza virus strains A/Puerto Rico/8/1934 (H1N1; A/PR8) and A/Philippines/82 (H3N2 subtype) were grown in embryonated hen's eggs as described [42]–[45] and used in this study to infect mice.

### Treatment of Mice with DK119 and Influenza Virus A/PR8

Female BALB/c mice (6–8 weeks old, Harlan Laboratories) were used (n = 5–6 each group). For oral administration of DK119, mice received intra-gastric administration of 200 µl of suspension containing 10^9^ or 10^8^ CFU of live DK119 once daily for consecutive 10 days via a feeding needle. At the 10^th^ day of DK119 oral treatment, mice were infected with 50 µl of influenza virus A/PR8 virus (2.5 LD_50_, 50% lethal dose for BALB/c mice) as described [42]–[45]. Oral administration was continued until 14 days after infection, and body weight changes and survival rates were monitored daily for 2 weeks. A control group was infected with the same dose of A/PR8 virus without DK119 probiotic. For intranasal treatment, mice were anesthetized with isoflurane using an oxygen controlled machine (Baxter, Deerfield, IL) and then administered DK119 (10^9^, 10^8^, or 10^7^ CFU per mouse) and A/PR8 H1N1 virus (2.5 LD_50_) or A/Philippines/82 H3N2 virus (2 LD_50_). All animals that were infected with virus were daily monitored, and their weight and survival rates were recorded. The humane endpoint for survival studies is 25% loss in body weights and mice were humanely euthanized to avoid severe illness. Animal experiments presented in this study were approved by the Georgia State University IACUC review boards (IACUC A11026). All animal experiments and husbandry have been carried out under the guidelines of the Georgia State University IACUC. Georgia State University IACUC operates under the federal Animal Welfare Law (administered by the USDA) and regulations of the Department of Health and Human Services.

### Lung Viral Titers and Cytokine Assays

To collect lung samples, mice were killed at day 4 post infection with A/PR8 virus, and bronchoalveolar lavage fluids (BALF) and lung samples were collected from individual mice as described [43]–[45]. Lung extracts were prepared using a mechanical tissue grinder with 1.5 ml of PBS per each lung and egg-infectious viral titers were determined by hemagglutination activity assay in the 10-day-old embryonated chicken eggs [46]. Levels of cytokines in BALF were determined by cytokine ELISA as described [42]–[45], and Ready-Set-Go IL-4, IL-6, IL-12, TNF-α, IFN-γ cytokine kits (eBioscience, San Diego, CA) were used following the manufacturer’s procedure.

### Treatment of Mice with Clodronate-liposomes and Flow Cytometry

Liposome-encapsulated clodronate liposomes (Foundation Clodronateliposomes.com, The Netherlands) were prepared as previously described [47]. Four hours prior to infection with influenza virus A/Philippines/82 (H3N2), a group of DK119-treated mice (n = 6 BALB/c) was intranasally administered clodronate-liposomes (100 µl per mouse) to deplete dendritic and macrophage cells [31], [32], [48]. Clodronate was a kind gift of Roche Diagnostics GmbH, Mannheim, Germany. To determine the efficacy of alveolar macrophage depletion, lung and bronchoalveolar cells were obtained at day 4 post treatment with clodronate and stained with surface marker antibodies (CD45, CD3, F4/80, CD11b, CD11c). All flow cytometry antibodies were purchased from BD Biosciences or eBiosciences. Stained cells were acquired by flow cytometer LSRFortessa (BD Biosciences) and analyzed using the FlowJo program (Tree Star Inc.) to confirm the phenotypes of cells depleted.

### Histology

Lung samples were collected after blood taken via the abdominal aorta, fixed in 10% neutral buffered formalin for 48 hrs, transferred to 70% ethanol, and embedded in paraffin. Finally, sectioned lung tissues were stained with hematoxylin and eosin and then examined under the microscope as described [49]–[51].

### Bone Marrow Derived Dendritic Cell (BMDC) Preparation

Dendritic cells (BMDCs) were generated from bone marrow of BALB/c mice [52]–[54]. Briefly, the harvested cells from femur and tibia bones were seeded onto 6-well culture plates and incubated in the presence of granulocytes-macrophages colony stimulation factor (10 ng/ml). BMDCs (2.5×10^5^ cells/ml) were treated with live DK119, harvested after 2 days’ incubation, and stained with CD11c dendritic cell marker antibody.

### Statistical Analysis

To determine the statistical significance among multiple groups, an ANOVA test was used in the GraphPad Prism program whereas a two-tailed Student’s t-test was used when comparing two different conditions. A p value less than 0.05 was considered to be significant.
